# The epigenetic signature of systemic insulin resistance in obese women

**DOI:** 10.1007/s00125-016-4074-5

**Published:** 2016-08-18

**Authors:** Peter Arner, Anna-Stina Sahlqvist, Indranil Sinha, Huan Xu, Xiang Yao, Dawn Waterworth, Deepak Rajpal, A. Katrina Loomis, Johannes M. Freudenberg, Toby Johnson, Anders Thorell, Erik Näslund, Mikael Ryden, Ingrid Dahlman

**Affiliations:** 1 Department of Medicine, Karolinska Institutet, Karolinska University Hospital, C2:94, Huddinge, S-141 86 Stockholm, Sweden; 2 GlaxoSmithKline R&D, Stevenage, UK; 3 Department of Biosciences and Nutrition, Karolinska Institutet, Stockholm, Sweden; 4 GlaxoSmithKline R&D, Research Triangle Park, NC USA; 5 Computational and Systems Biology, Discovery Sciences, Janssen Pharmaceutical, Research & Development, LLC, San Diego, CA USA; 6 GlaxoSmithKline R&D, King of Prussia, PA USA; 7 Pfizer Worldwide Research and Development, Groton, CT USA; 8 Department of Surgery, Ersta Hospital, Stockholm, Sweden; 9 Department of Clinical Sciences, Karolinska Institutet, Danderyd Hospital, Danderyd, Sweden

**Keywords:** CpG island, DNA methylation, Visceral adipose tissue

## Abstract

**Aims/hypothesis:**

Insulin resistance (IR) links obesity to type 2 diabetes. The aim of this study was to explore whether white adipose tissue (WAT) epigenetic dysregulation is associated with systemic IR by genome-wide CG dinucleotide (CpG) methylation and gene expression profiling in WAT from insulin-resistant and insulin-sensitive women. A secondary aim was to determine whether the DNA methylation signature in peripheral blood mononuclear cells (PBMCs) reflects WAT methylation and, if so, can be used as a marker for systemic IR.

**Methods:**

From 220 obese women, we selected a total of 80 individuals from either of the extreme ends of the distribution curve of HOMA-IR, an indirect measure of systemic insulin sensitivity. Genome-wide transcriptome and DNA CpG methylation profiling by array was performed on subcutaneous (SAT) and visceral (omental) adipose tissue (VAT). CpG methylation in PBMCs was assayed in the same cohort.

**Results:**

There were 647 differentially expressed genes (false discovery rate [FDR] 10%) in SAT, all of which displayed directionally consistent associations in VAT. This suggests that IR is associated with dysregulated expression of a common set of genes in SAT and VAT. The average degree of DNA methylation did not differ between the insulin-resistant and insulin-sensitive group in any of the analysed tissues/cells. There were 223 IR-associated genes in SAT containing a total of 336 nominally significant differentially methylated sites (DMS). The 223 IR-associated genes were over-represented in pathways related to integrin cell surface interactions and insulin signalling and included *COL5A1*, *GAB1*, *IRS2*, *PFKFB3* and *PTPRJ*. In VAT there were a total of 51 differentially expressed genes (FDR 10%); 18 IR-associated genes contained a total of 29 DMS.

**Conclusions/interpretation:**

In individuals discordant for insulin sensitivity, the average DNA CpG methylation in SAT and VAT is similar, although specific genes, particularly in SAT, display significantly altered expression and DMS in IR, possibly indicating that epigenetic regulation of these genes influences metabolism.

**Electronic supplementary material:**

The online version of this article (doi:10.1007/s00125-016-4074-5) contains peer-reviewed but unedited supplementary material, which is available to authorised users.

## Introduction

The impaired ability of insulin to induce cellular responses (i.e. insulin resistance [IR]) is a pathophysiological mechanism that links obesity to metabolic disorders such as type 2 diabetes and cardiovascular disease [1]. Both genetic and epigenetic factors are implicated in the development of systemic IR [2], which may be characterised by elevated circulating levels of insulin in the fasting state despite normal or elevated glucose levels. The association between IR and excess abdominal fat, in particular in the intra-abdominal or visceral adipose tissue (VAT) depot, is believed to be mediated by increased spontaneous hydrolysis of lipids (i.e. adipocyte lipolysis) [3]. Released NEFA can induce IR in the liver [4]. In addition, systemic IR is characterised by ectopic triacylglycerol accumulation in skeletal muscle and the liver [5]. Other pathways implicated in systemic IR include low-grade inflammation in white adipose tissue (WAT) [6].

An unfavourable intrauterine environment is associated with IR in adulthood suggesting a, possibly epigenetically regulated, metabolic memory [7]. The term ‘epigenetics’ refers to stable long-term alterations in the transcriptional potential of cells and includes histone modifications and DNA methylation, the latter occurring mainly in the context of CG dinucleotides (CpGs) [8]. In any given individual, the epigenetic profiles can differ substantially between different organs and cell types [9]. In WAT, global as well as site-specific differences in CpG methylation have been associated with obesity and type 2 diabetes [10–12]. A recent epigenome-wide association study identified one locus where CpG methylation in CD4^+^ T cells is significantly associated with IR [13]. However, to our knowledge, no study of genome-wide CpG methylation profiling in the organs directly implicated in the development of IR has previously been reported.

The aim of this study was to explore whether systemic IR is associated with epigenetic dysregulation of WAT, determined by genome-wide CpG methylation and gene expression profiling in subcutaneous adipose tissue (SAT) and VAT. Adipose tissue is not ideal for routine clinical examinations; therefore, a secondary aim was to determine whether the DNA methylation signature in peripheral blood mononuclear cells (PBMCs) reflects WAT methylation and may thus be used as a marker for systemic IR.

## Methods

### Participants and clinical evaluation

The 80 women included in this study were selected from the extremes of insulin sensitivity, as measured by HOMA-IR [14], from 220 obese women who participated in a clinical trial on the effect of bariatric surgery (ClinicalTrial.gov registration no. NCT01785134). The sample size was selected based on previous experience from transcriptome and DNA methylation profiling on WAT in relation to clinical metabolic phenotypes [10]. Of the 80 women, none had undergone any active weight-reducing attempt for at least 6 months prior to surgery. Eight women were diagnosed with hypertension, seven of which were prescribed antihypertensive treatment (ACE inhibitors, *n* = 3; diuretics, *n* = 2; calcium-channel blockers, *n* = 2; β-blockers, *n* = 5). Eleven patients were prescribed antidepressants, and one patient was taking methylphenidate for attention deficit hyperactivity disorder. Mild impaired kidney function (*n* = 1), obstructive sleep apnoea (*n* = 1), von Willebrand’s disease (*n* = 1) and substituted vitamin B12 deficiency (*n* = 1) were each diagnosed. Otherwise, participants were healthy according to medical history. All sampling and measurements were performed before or during bariatric surgery (laparoscopic gastric bypass).

Participants were investigated at 08:00 hours after an overnight fast. Anthropometric measurements were performed followed by venous blood sampling. Blood glucose and lipids were analysed at the Karolinska University hospital’s routine chemistry laboratory (Stockholm, Sweden). Plasma insulin was measured by ELISA (Mercodia, Uppsala, Sweden) as previously described [15]. Insulin sensitivity was assessed by HOMA-IR and was calculated from fasting measures of glucose and insulin as described [14]. High HOMA-IR values indicate IR. The 40 women with the highest HOMA-IR values and the 40 women with the lowest values were selected for inclusion in the present study. PBMCs were isolated in BD Vacutainer Cell Preparation tubes (Becton, Dickinson San Jose, CA, USA) and stored as pellets at −80°C for further analysis.

The study was approved by the Regional Ethics Committee in Stockholm and all participants gave their written informed consent prior to participation. The study was carried out in accordance with the principles of the Declaration of Helsinki as revised in 2008.

### WAT sampling

Biopsies from the abdominal SAT depot were obtained from the surgical incision. Omental adipose tissue (visceral adipose tissue [VAT]) specimens were obtained using ultrasound scissors immediately after surgeons entered the abdominal cavity. Participants were fasted overnight and 154 mmol/l NaCl was given by i.v. infusion until adipose tissue specimens were removed. All WAT samples were rapidly rinsed in NaCl (154 mmol/l) and specimens of 300 mg unfractionated WAT were immediately frozen in liquid nitrogen and kept at −70°C for subsequent DNA and RNA preparation.

### Global transcriptome assays

From high-quality total RNA we prepared and hybridised biotinylated complementary RNA to GeneChip Human Transcriptome Arrays 2.0 (HTA; Affymetrix, Santa Clara, CA, USA) as described in the electronic supplementary material (ESM) Methods. Of the 23,442 probesets annotated with a gene symbol, 5860 (25%) transcripts with the lowest mean expression and 5860 (25%) with the lowest variation in expression (i.e. SD divided by mean expression) were excluded, resulting in 11,722 probesets being taken forward for subsequent analysis of differentially expressed genes. The applied cut-off for mean expression was used to exclude a set of organ-specific genes that should not be expressed in adipose tissue according to the literature. Webgestalt (http://bioinfo.vanderbilt.edu/webgestalt/) was used to identify pathways over-represented among differentially expressed genes and differentially methylated sites (DMS) [16].

### DNA methylation microarray assays

DNA extracted from SAT and VAT pieces, as well as from PBMCs, was assayed using the Infinium Human Methylation 450 (450 K) BeadChips (Illumina, San Diego, CA, USA) as described in ESM Methods [17]. BeadChip images were processed as described in ESM Methods. For differential methylation analysis, β values were converted to *M* values (*M* = Log_2_[β/(1 − β)]), which have a more appropriate distribution for statistical tests for comparisons between groups. Before analysis of DMS a number of filtering steps were performed resulting in 112,057 (SAT), 124,089 (VAT) and 99,462 (PBMCs) probes, respectively, being taken forward to identify DMS.

Methylation data have been deposited in the National Center for Biotechnology Information Gene Expression Omnibus (GEO; http://ncbi.nlm.nih.gov/geo, accession number GSE76399).

### Validation experiments

Ten differentially expressed genes with DMS in SAT were selected for validation experiments. The genes were selected because they displayed consistent results in SAT and either VAT or PBMCs, or because they were mentioned in the Discussion. Gene expression was measured by quantitative real time-PCR (qPCR) using recommended inventoried Taqman assays from Applied Biosystems (Thermo Fisher Scientific, Waltham, MA, USA). Each sample was analysed once. Group assignment was blinded during experimentation.

Eleven DMS in SAT, in seven genes, were selected for validation by EpiTYPER (Agena Biosciences, San Diego, CA, USA), see ESM Methods for details. We were unable to design EpiTYPER assays for DMS in some differentially expressed genes validated by qPCR. We therefore selected a DMS *COL4A1* for confirmation although this gene was not quantified by qPCR.

### Statistical analysis

We used the Bioconductor package, Limma (https://bioconductor.org/packages/release/bioc/html/limma.html) to analyse the methylation *M* values to identify DMS between insulin-resistant and insulin-sensitive women, adjusting for BMI and age [18–20]. A threshold of *p* < 0.05 was used in the epigenetic analysis. We also used parametric analysis in Limma to compare gene expression levels (Log_2_) between the insulin-resistant and insulin-sensitive groups adjusting for BMI. In transcriptome analysis a thresholds false discovery rate (FDR) of 10% was used. A *t* test was applied to compare clinical phenotypes, average global DNA methylation and validation results (qPCR and EpiTYPER) between the insulin-resistant and insulin-sensitive groups; a χ^2^ test was used to compare proportions.

## Results

### Clinical characteristics of participants

The clinical characteristics of the included participants are detailed in Table 1. As expected from the study design, the insulin-resistant group had substantially higher HOMA-IR, fasting plasma glucose and fasting serum insulin as compared with the insulin-sensitive group. The insulin-resistant group also displayed higher body weight, BMI, waist circumference and plasma triacylglycerol concentrations. Total and HDL-cholesterol levels were similar and there was no significant difference in age when comparing the groups. Thus, the groups were representative of the insulin-resistant or insulin-sensitive state.

**Table 1:** Clinical characteristics of cohort

Characteristic	Insulin resistant (*n* = 40)	Insulin sensitive (*n* = 40)	*p* value
Age (years)	36.4 ± 6.3	35.7 ± 5.7	0.57
Weight (kg)	116.8 ± 16.7	110.1 ± 11.7	0.04
BMI (kg/m^2^)	42.7 ± 4.7	39.1 ± 3.0	8.37 × 10^−5^
Waist circumference (cm)	129.8 ± 11.9	122.3 ± 11.1	0.0061
fP Glucose (mmol/l)	6.0 ± 1.3	5.1 ± 0.4	9.07 × 10^−5^
fS Insulin (pmol/l)	127 ± 39	29 ± 8	1.29 × 10^−25^
HOMA-IR	5.6 ± 2.0	1.1 ± 0.3	7.11 × 10^−23^
fS Cholesterol (mmol/l)	4.6 ± 1.1	4.5 ± 0.9	0.64
fS HDL-cholesterol (mmol/l)	1.1 ± 0.4	1.2 ± 0.3	0.78
fS Triacylglycerols (mmol/l)	1.45 ± 0.7	1.02 ± 0.4	0.000786

Data are means ± SD; all participants are women

Groups were compared with *t* test

fP, fasting plasma; fS, fasting serum

### Transcriptome profile in SAT and VAT

Comparison of the expression levels of 11,722 transcripts between insulin-resistant and insulin-sensitive women adjusted for BMI identified 647 differentially expressed genes in SAT (represented by 656 probesets, FDR 10% [see ESM Table 1]). Expression of ten differentially expressed genes in SAT was confirmed by qPCR; all displayed directionally consistent results between insulin-resistant and insulin-sensitive women in both microarray and qPCR analysis, of which eight genes remained nominally significant with qPCR (ESM Table 2). We compared these results with previously reported genome-wide transcriptome analyses of SAT between insulin-resistant and insulin-sensitive individuals according to HOMA-IR. Among 321 differentially expressed genes in SAT of 40 European-Americans, reported by Elbein et al (FDR 5%) [21], 26 genes overlapped with the present study, all of which displayed directionally consistent change in expression (*p* < 3.4 × 10^−7^). Among 373 differentially expressed genes in SAT (top/bottom 20%) from 323 individuals, reported by Qatanani et al [22], 19 genes overlapped with the present study and 18 of these displayed directional consistency (*p* < 9.6 × 10^−5^) (ESM Table 1).

The 647 differentially expressed genes were over-represented for a number of pathways (Table 2), including pathways related to inflammation and immunity (e.g. TNF-related apoptosis-inducing ligand [TRAIL] signalling, IL3-mediated signalling and vascular endothelial growth factor receptor [VEGFR] signalling), which is in agreement with the findings by Elbein et al [21] and Qatanani et al [22]. As expected, genes in the insulin signalling pathway were also over-represented. The 70 differentially expressed genes in the insulin signalling pathway are shown in ESM Table 3 and include *IRS2*, which was downregulated by 15%, and *IL6R*, which was upregulated by 7% in insulin-resistant women.

**Table 2:** Over-representation of specific gene-sets among differentially expressed genes in SAT between insulin-resistant and insulin-sensitive women^a^

Pathway^c^	Observed^b^	Expected^b^	Adjusted *p* value
TRAIL signalling pathway	73	49	0.0024
Signalling events mediated by VEGFR1 and VEGFR2	70	48	0.0024
GMCSF-mediated signalling events	70	48	0.0024
IL3-mediated signalling events	70	48	0.0024
PAR1-mediated thrombin signalling events	70	48	0.0024
S1P1 pathway	70	47	0.0024
IFN-γ pathway	70	48	0.0024
ErbB1 downstream signalling	70	47	0.0024
β_1_ integrin cell surface interactions	78	50	0.0024
Urokinase-type plasminogen activator and uPAR-mediated signalling	70	47	0.0024
Plasma membrane oestrogen receptor signalling	71	48	0.0024
IGF1 pathway	70	47	0.0024
Insulin pathway	70	47	0.0024
Arf6 signalling events	70	47	0.0024

^a^Webgestalt was used to identify over-represented gene-sets (Pathway commons) among 647 differentially expressed genes as compared with all 11,722 analysed genes using default settings

^b^Number of differentially expressed genes

Arf6, ADP-ribosylation factor 6; ErbB1, epidermal growth factor receptor; GMCSF, granulocyte-macrophage colony-stimulating factor; PAR1, proteinase-activated receptor 1; S1P1, sphingosine-1-phosphate receptor; uPAR, plasminogen activated receptor urokinase type

In VAT there were 51 differentially expressed genes (represented by 52 probesets) between insulin-resistant and insulin-sensitive women at FDR 10% (Table 3). For comparison, Qatanani et al [22] reported 788 differentially expressed genes in VAT between insulin-resistant and insulin-sensitive individuals (top/bottom 20%), out of which eight genes overlapped with the 51 differentially expressed genes in the present study (i.e. *GSDMB* [fold changes insulin-resistant vs insulin-sensitive: 0.82], *AGPAT9* [0.78], *PAIP2B* [0.85], *CA3* [0.45], *SERPINI1* [0.91], *RASSF4* [1.13], *MYD88* [1.09], *SLCO2B1* [1.24]); all eight genes displayed directionally consistent expression in both studies (*p* < 4.7 × 10^−3^) [22] (Table 3). The 51 differentially expressed genes in VAT in our study were not over-represented for any specific pathway.

**Table 3:** Differentially expressed genes in VAT between insulin-resistant and insulin-sensitive women

Probeset	Gene	VAT				VAT^a^	SAT		
		IR	IS	IR/IS	Adjusted *p* value^b^	IR/IS	IS	IR/IS	Adjusted *p* value^b^
TC09001184.hg.1	*PGM5-AS1*	174 (27)	219 (34)	0.79	0.002		127	0.87	
TC09001281.hg.1	*GKAP1*	56 (4)	63 (6)	0.89	0.002		56	0.90	0.021
TC17002851.hg.1	*GSDMB*	92 (10)	112 (19)	0.82	0.0082	0.84	91	0.84	0.016
TC04000460.hg.1	*AGPAT9*	70 (12)	89 (18)	0.78	0.022	0.79	55	1.00	
TC12000227.hg.1	*PDE3A*	109 (22)	133 (22)	0.82	0.028		100	0.82	0.033
TC09001585.hg.1	*SCAI*	62 (5)	69 (7)	0.90	0.034		67	0.88	0.016
TC15000030.hg.1	*GOLGA8IP*	195 (17)	217 (22)	0.90	0.034		186	0.93	0.08
TC05000782.hg.1	*ARHGAP26*	127 (18)	109 (13)	1.16	0.034		111	1.09	0.078
TC22000816.hg.1	*ST13*	499 (38)	558 (57)	0.89	0.035		537	0.89	0.016
TC20000575.hg.1	*SIGLEC1*	189 (30)	164 (18)	1.15	0.035		164	1.11	0.072
TC09000495.hg.1	*ANP32B*	215 (14)	237 (21)	0.91	0.038		244	0.92	0.019
TC15000157.hg.1	*GOLGA8J*	228 (24)	257 (28)	0.89	0.038		203	0.91	0.03
TC05000212.hg.1	*ISL1*	80 (10)	99 (25)	0.80	0.041		29	0.99	
TC02001974.hg.1	*PAIP2B*	96 (10)	114 (17)	0.85	0.042	0.93	87	0.89	
TC15002013.hg.1	*TARSL2*	79 (4)	86 (7)	0.92	0.043		83	0.94	0.078
TC05001954.hg.1	*FAT2*	56 (7)	49 (5)	1.14	0.043		76	1.11	
TC01003789.hg.1	*ST13P19*	52 (5)	58 (6)	0.89	0.043		51	0.91	0.048
TC15002805.hg.1	*ULK4P1*	172 (38)	220 (50)	0.78	0.047		142	0.82	0.019
TC17001703.hg.1	*MBTD1*	106 (7)	116 (11)	0.91	0.048		109	0.92	0.031
TC20000926.hg.1	*KCNB1*	150 (20)	127 (23)	1.18	0.052		159	1.12	
TC06004132.hg.1	*MOCS1*	162 (33)	205 (40)	0.79	0.052		190	0.86	0.014
TC05001714.hg.1	*LOX*	206 (38)	169 (29)	1.22	0.052		291	1.07	
TC05001317.hg.1	*CCL28*	58 (4)	63 (6)	0.91	0.058		61	0.97	
TC07001811.hg.1	*AASS*	103 (11)	118 (16)	0.88	0.059		95	0.85	0.021
TC08002581.hg.1	*CA3*	110 (59)	242 (176)	0.45	0.062	0.42	99	0.81	
TC03000892.hg.1	*SERPINI1*	49 (5)	54 (6)	0.91	0.062	0.81	36	0.95	
TC11000898.hg.1	*NAALAD2*	39 (7)	48 (10)	0.80	0.066		54	0.74	0.019
TC15000160.hg.1	*ULK4P3*	147 (37)	188 (43)	0.78	0.07		115	0.80	0.02
TC01000619.hg.1	*CDKN2C*	110 (18)	134 (25)	0.82	0.072		134	0.92	
TC10000289.hg.1	*RASSF4*	142 (19)	126 (13)	1.13	0.072	1.23	153	1.12	0.059
TC19000034.hg.1	*CIRBP*	565 (44)	615 (54)	0.92	0.072		564	0.97	
TC18000224.hg.1	*PHLPP1*	91 (6)	101 (9)	0.91	0.072		105	0.89	0.03
TC13000436.hg.1	*UPF3A*	177 (13)	190 (16)	0.93	0.072		209	0.96	
TC04001410.hg.1	*ADH1B*	3013 (467)	3478 (495)	0.87	0.074		3236	0.80	0.017
TC15001546.hg.1	*DAPK2*	146 (21)	171 (26)	0.86	0.074		159	0.85	0.0088
TC04001305.hg.1	*CXCL10*	60 (57)	36 (11)	1.67	0.074		57	0.99	
TC09000319.hg.1	*TJP2*	166 (14)	179 (14)	0.93	0.074		194	0.98	
TC03000187.hg.1	*MYD88*	172 (16)	158 (14)	1.09	0.076	1.12	193	1.06	
TC07001493.hg.1	*GTF2IRD2P1*	151 (14)	163 (15)	0.92	0.081		158	0.92	
TC02002891.hg.1	*ARL4C*	77 (9)	69 (10)	1.12	0.081		61	1.09	
TC09002904.hg.1	*NIPSNAP3B*	78 (20)	102 (28)	0.77	0.081		96	0.77	0.019
TC12001300.hg.1	*ABCC9*	338 (63)	391 (63)	0.86	0.082		630	0.75	0.0026
TC12001299.hg.1	*KCNJ8*	129 (11)	142 (14)	0.91	0.088		144	0.89	0.02
TC11000933.hg.1	*CEP57*	98 (7)	107 (13)	0.92	0.089		110	0.89	0.019
TC11000802.hg.1	*SLCO2B1*	264 (66)	213 (52)	1.24	0.094	1.26	217	1.19	0.087
TC02000395.hg.1	*PNO1*	57 (5)	53 (4)	1.08	0.094		71	1.06	
TC01001043.hg.1	*PHGDH*	119 (15)	135 (22)	0.88	0.094		92	0.91	
TC11001197.hg.1	*ADAMTS15*	98 (12)	88 (10)	1.11	0.094		110	1.28	0.021
TC18000132.hg.1	*RNF125*	95 (10)	109 (15)	0.88	0.094		104	0.95	
TC02002086.hg.1	*ANKRD20A8P*	42 (6)	47 (7)	0.91	0.094		41	0.92	0.07
TC01001866.hg.1	*ADCK3*	181 (13)	201 (23)	0.90	0.095		192	0.93	0.071

Data are shown as average (SD) for VAT or average for SAT

^a^Comparison with published transcriptome profile [27] on VAT from insulin-resistant vs insulin-sensitive individuals

^b^Gene expression was compared between groups using Limma and adjusting for BMI; threshold FDR < 10%

IR, insulin-resistant; IS, insulin-sensitive

**Fig. 1:**
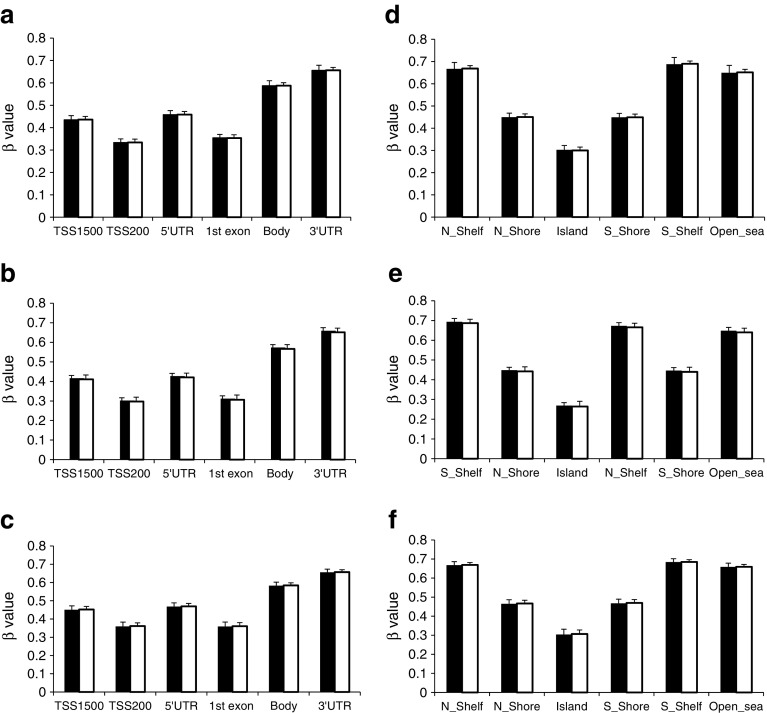
DNA methylation landscape in insulin-resistant vs insulin-sensitive women in SAT (**a**, **d**), VAT (**b**, **e**) and PBMCs (**c**, **f**). Based on Illumina annotation, 112,057 (SAT), 124,089 (VAT) and 99,462 (PBMCs) CpG probes were mapped to genome regions. We calculated the average level of DNA methylation within each of the insulin-resistant (black bars) and insulin-sensitive (white bars) groups stratified on genome region in relation to functional gene regions (**a**, **b**, **d**) and CpG content (**d**, **e**, **f**). TSS1500, within 1500 bp of transcriptional start site (TSS); TSS200, within 200 bp of TSS. Genome locations: Island, CpG island; N_Shelf, upstream CpG island shelf; N_Shore, upstream CpG island shore; S_Shore, downstream CpG island shore; S_Shelf, downstream CpG island shelf; Open_sea; other CpG regions

To assess possible depot-specific differences in gene expression, we overlapped the gene array data from VAT and SAT. ESM Fig. 1 a shows a histogram of the per-gene correlation between gene expression in VAT and SAT tissue samples and Fig. 1b shows a boxplot of between-sample correlation. As expected, within-participant correlation is higher than between-participant. All 51 differentially expressed genes in VAT displayed directionally consistent differences in expression in SAT between insulin-resistant and insulin-sensitive women, and 30 of these genes were significant (FDR 10%; Table 3). Conversely, of the 647 differentially expressed genes in SAT, all displayed directionally consistent differences in VAT (ESM Table 1), 209 of which were nominally significant (*p* ≤ 0.05). The magnitude of the difference in expression of these genes between insulin-resistant and insulin-sensitive women was comparable between VAT (median difference in expression 8.8%; range 3.8–23.9%) and SAT (median 10.7%; range 4.6–38.5%). For individual genes, the median difference in ratio of expression between insulin-resistant and insulin-sensitive women was 0.027% (range 0.005–23.0%) between adipose depots. Together, these comparisons suggest that in the present cohort, IR is associated with similar dysregulations of gene expression in the examined WAT depots.

### Global pattern of CpG methylation

The average degree of DNA methylation (i.e. the average β value for all probes remaining after filtering) was compared between the insulin-resistant and insulin-sensitive groups. There were no significant differences in either SAT (insulin-resistant 0.504 ± 0.019 [average β value ± SD]); insulin-sensitive 0.507 ± 0.013), VAT (insulin-resistant 0.483 ± 0.014; insulin-sensitive 0.477 ± 0.022) or PBMCs (insulin-resistant 0.508 ± 0.020; insulin-sensitive 0.510 ± 0.015). The average level of DNA methylation stratified by genome region in relation to CpG content and functional parts of genes is shown in Fig. 1.

### DMS in SAT

Comparison of CpG methylation in SAT between insulin-resistant and insulin-sensitive women was assessed at 112,057 sites. Although none of the DMS were significant after FDR correction, 10,746 were nominally significant with median differences in methylation of 0.024 (range 4 × 10^−4^ to 0.092) between groups (*p* ≤ 0.05). These data were compared with results from other DNA methylation profiling studies on SAT applying the same 450 K platform. Nilsson et al reported, in a cohort of 56 individuals, 15,627 DMS (*q* < 0.15) in WAT associated with type 2 diabetes [10]; 671 of the DMS overlapped with those in the present study, of which 592 displayed directionally consistent differences in methylation in both cohorts (*p* < 2.7 × 10^−87)^ (ESM Table 4) [10]. In a study of 190 men and women, Rönn et al identified 39,533 CpG sites whose methylation in WAT of women was associated with BMI. Of these BMI-associated CpG sites, 2052 overlapped with the present study and 1973 displayed directionally consistent differences in methylation (*p* < 1 × 10^−90^) (ESM Table 4) [20]. Benton et al reported 3601 DMS before vs after weight loss induced by bariatric surgery [12]. Ninety-three DMS overlapped with the present study out of which 91 sites displayed directionally consistent results between obese individuals before weight loss and insulin-resistant individuals (*p* < 2.7 × 10^−20^) (ESM Table 4). Eleven DMS were confirmed by EpiTYPER; nine displayed directionally consistent results between insulin-resistant and insulin-sensitive women in both microarray and EpiTYPER analysis, of which four remained nominally significant, and three more were close to significance (*p* < 0.06) (ESM Table 2). It is worth noting that, of the DMS analysed by EpiTYPER, seven had been previously reported, all of which were confirmed by the present study.

**Fig. 2:**
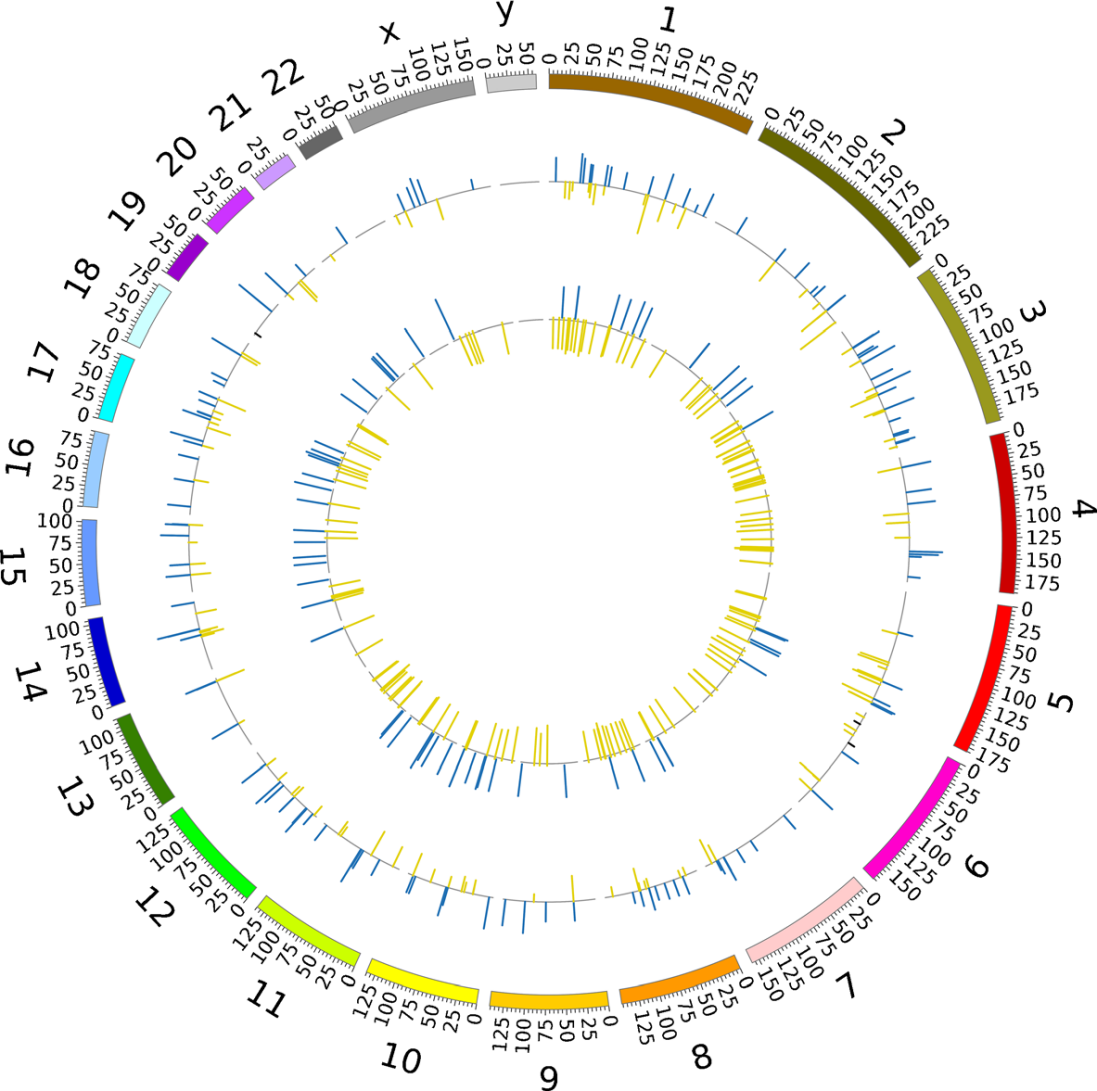
Chromosomal position of 223 IR-associated genes containing a total of 336 DMS. Inner circle shows gene expression data (blue, upregulated expression in IR; yellow, downregulated expression in IR), outer circle represents methylation data (blue, high methylation in IR; yellow, low methylation in IR)

Next, we merged the 647 differentially expressed genes in SAT with the 10,746 DMS and identified 223 IR-associated genes containing a total of 336 DMS (ESM Table 5). These genes are evenly distributed in the genome, and each gene contains one or a few DMS (Fig. 2). A subset of these genes is listed in Table 4. Twenty-nine genes displayed direct, positive or negative, correlation between gene expression and methylation (ESM Table 6). Whereas CpG methylation in 5’ regions of genes has classically been associated with reduced gene expression, CpG methylation in gene bodies has been reported to stimulate gene expression [23]. It was therefore of interest to map the IR-associated DMS in relation to gene region, and relate the degree of methylation to gene expression. Among 158 DMS in 5’ regions of genes, 67 CpG sites displayed reciprocal direction of effect between gene expression and CpG methylation. Among 178 DMS in gene bodies and 3’ untranslated regions (3’UTRs), 80 CpG sites displayed a positive association between changes in DNA methylation and gene expression. Thus, there was no evidence that DNA methylation in the 5’ regions of genes preferentially repressed gene expression, nor the opposite in gene bodies.

**Table 4:** A subset of differentially expressed genes accompanied by DMS in SAT between insulin-resistant vs insulin-sensitive women^a^

Probe	Gene	Relation to gene region	DNA methylation						Gene expression		
			IS average	IR − IS	*p* value	T2D^b,c^ [10]	BMI^b,d^ [20]	GBP^b,e^ [12]	IS average	IR/IS	*p* value
cg07251857	ALPK3	1st exon	0.546	0.026	0.022		0.016		76	0.89	2.56 × 10^−3^
cg06532379	*ALPK3*	1st exon	0.193	0.039	0.015		0.015		76	0.89	2.56 × 10^−3^
cg14080050	*B4GALT1*	Body	0.447	−0.037	0.015		−0.014		228	1.10	1.48 × 10^−3^
cg13858803	*B4GALT1*	Body	0.566	0.027	0.040		0.027		228	1.10	1.48 × 10^−3^
cg00300298	*BCL2L1*	Body	0.251	−0.037	0.038		−0.019		151	1.07	2.01 × 10^−3^
cg12873919	*BCL2L1*	Body	0.504	−0.036	0.032				151	1.07	2.01 × 10^−3^
cg03290977	*C1QTNF7*	Body	0.247	−0.035	0.034		−0.024		44	0.86	3.28x10^−3^
cg01939704	*C1QTNF7*	Body	0.616	0.020	0.022				44	0.86	3.28 × 10^−3^
cg07538039	*C1QTNF7*	Body	0.610	0.025	0.021				44	0.86	3.28 × 10^−3^
cg06097727	*C1QTNF7*	Body	0.547	0.035	0.043		0.016		44	0.86	3.28 × 10^−3^
cg24829483	*C1QTNF7*	5′UTR	0.633	0.039	0.034				44	0.86	3.28 × 10^−3^
cg00545229	*C1QTNF7*	TSS200	0.563	0.041	0.018				44	0.86	3.28 × 10^−3^
cg15372098	*C3orf26*	Body	0.027	−0.016	0.014				69	0.92	3.98 × 10^−4^
cg00991994	*C3orf26*	Body	0.401	0.055	0.039	0.067	0.035		69	0.92	3.98 × 10^−4^
cg17351376	*CD248*	1st exon	0.504	0.019	0.032				239	1.42	1.03 × 10^−3^
cg07145284	*CD248*	TSS200	0.085	0.029	0.038		0.018		239	1.42	1.03 × 10^−3^
cg00350296	*CD248*	TSS1500	0.158	0.041	0.018		0.022		239	1.42	1.03 × 10^−3^
cg13860849	*CD248*	1st exon	0.191	0.054	0.002		0.015		239	1.42	1.03 × 10^−3^
cg10772263	*CHST3*	5′UTR	0.322	0.020	0.028		0.025		113	1.17	1.31 × 10^−3^
cg04268405	*CHST3*	TSS1500	0.369	0.042	0.024			0.219	113	1.17	1.31 × 10^−3^
cg12081643	*COL4A1*	3′UTR	0.670	−0.042	0.008				530	1.17	1.34 × 10^−3^
cg20818806	*COL4A1*	Body	0.299	0.042	0.019				530	1.17	1.34 × 10^−3^
cg02658690	*COL4A1*	Body	0.207	0.042	0.014			0.218	530	1.17	1.34 × 10^−3^
cg10908116	*COL4A1*	Body	0.247	0.043	0.017	0.053	0.026		530	1.17	1.34 × 10^−3^
cg02099572	*COL4A1*	Body	0.140	0.047	0.005	0.056			530	1.17	1.34 × 10^−3^
cg03430597	*COL5A1*	Body	0.751	0.018	0.004		0.018		162	1.10	5.97 × 10^−4^
cg24354213	*COL5A1*	Body	0.601	0.027	0.023		0.014		162	1.10	5.97 × 10^−4^
cg14274542	*COL5A1*	Body	0.596	0.037	0.019		0.012		162	1.10	5.97 × 10^−4^
cg10765212	*COL5A2*	TSS200	0.129	0.021	0.047				246	1.20	3.25 × 10^−4^
cg15194531	*FMNL1*	Body	0.466	0.041	0.005		0.018		165	1.09	5.32 × 10^−4^
cg08145262	*FRS2*	5′UTR	0.658	0.031	0.020		0.020		155	0.93	1.64 × 10^−3^
cg19563525	*FRS2*	5′UTR	0.382	0.035	0.006		0.017		155	0.93	1.64 × 10^−3^
cg10227830	*GAB1*	Body	0.272	0.039	0.016				141	0.89	1.53 × 10^−4^
cg25911551	*GAB1*	Body	0.494	0.046	0.049		0.019		141	0.89	1.53 × 10^−4^
cg08202226	*GATAD2B*	TSS1500	0.793	−0.057	0.018		−0.029		282	0.94	3.97 × 10^−3^
cg05514401	*IRS2*	1st exon	0.792	0.031	0.002		0.028		242	0.85	1.24 × 10^−3^
cg11624345	*KCNN4*	Body	0.391	0.025	0.025		0.014		87	1.06	4.11 × 10^−3^
cg03731131	*KCNN4*	Body	0.378	0.032	0.039				87	1.06	4.11 × 10^−3^
cg22904711	*KCNN4*	Body	0.313	0.060	0.002	0.047	0.015		87	1.06	4.11 × 10^−3^
cg14616541	*MYH10*	Body	0.834	0.024	0.010				292	0.87	1.64 × 10^−3^
cg22588546	*MYH10*	Body	0.496	0.047	0.008	0.039			292	0.87	1.64 × 10^−3^
cg21542094	*PFKFB3*	TSS1500	0.081	−0.001	0.025		−0.013		542	0.80	5.53 × 10^−5^
cg00902516	*PFKFB3*	Body	0.739	0.020	0.019		0.016		542	0.80	5.53 × 10^−5^
cg03261682	*PFKFB3*	Body	0.780	0.028	0.006		0.026		542	0.80	5.53 × 10^−5^
cg05686026	*PFKFB3*	Body	0.683	0.045	0.001		0.033		542	0.80	5.53 × 10^−5^
cg03478610	*PPP2R3A*	5′UTR	0.871	−0.031	0.034		−0.014		91	0.93	1.49 × 10^−3^
cg00369142	*PPP2R3A*	3′UTR	0.378	0.044	0.013	0.060	0.025		91	0.93	1.49 × 10^−3^
cg11468953	*PTPRJ*	Body	0.519	−0.039	0.027		−0.020		139	1.18	3.07 × 10^−3^
cg12124589	*QSOX1*	Body	0.775	−0.032	0.027		−0.020		175	1.08	1.70 × 10^−3^
cg09505809	*QSOX1*	TSS1500	0.179	0.039	0.031				175	1.08	1.70 × 10^−3^
cg00971364	*RBMS3*	TSS200	0.043	−0.018	0.034				381	0.90	5.14 × 10^−4^
cg23537305	*RBMS3*	Body	0.819	0.016	0.045		0.017		381	0.90	5.14 × 10^−4^
cg20299414	*RBMS3*	Body	0.729	0.035	0.018		0.013		381	0.90	5.14 × 10^−4^
cg27569887	*RBMS3*	3′UTR	0.698	0.043	0.026				381	0.90	5.14 × 10^−4^
cg16572224	*SH3PXD2B*	Body	0.816	−0.049	0.002	−0.039	−0.019		145	1.13	4.76 × 10^−3^
cg05223396	*SH3PXD2B*	Body	0.404	0.025	0.049				145	1.13	4.76 × 10^−3^
cg09744420	*STX11*	Body	0.654	0.041	0.002		0.020		356	0.89	2.98 × 10^−3^
cg19841369	*SYNE2*	Body	0.159	0.028	0.044		0.016		236	0.89	2.18 × 10^−3^
cg16725974	*SYNE2*	5′UTR	0.532	0.046	0.027	0.057	0.022		236	0.89	2.18 × 10^−3^
cg23250157	*SYNE2*	Body	0.756	0.061	0.018				236	0.89	2.18 × 10^−3^
cg18837713	*ZDHHC17*	Body	0.616	0.045	0.010		0.027		161	0.94	5.15 × 10^−3^

^a^Differentially expressed genes (10% FDR) accompanied by DMS (*p* < 0.05) in SAT between insulin-resistant and insulin-sensitive women. Groups were compared using Limma and adjusting for BMI (gene expression, DMS) and age (DMS). This table contains a subset of the ESM Table 4 and focuses on DMS confirmed from the literature and mentioned in the discussion

^b^Comparison with published transcriptome profiles on SAT from insulin-resistant vs insulin-sensitive individuals

^c^T2D vs control

^d^Regression coefficient

^e^After vs before bariatric surgery and weight loss

IR, insulin-resistant; IS, insulin-sensitive; T2D, type 2 diabetes

The 223 IR-associated genes were over-represented for pathways related to integrin cell surface interactions, focal adhesion and insulin signalling (ESM Table 7). Data for the insulin signalling genes are shown in Table 5.

**Table 5:** Differentially expressed insulin signalling pathway genes accompanied by DMS in SAT between insulin-resistant and insulin-sensitive women^a^

Probe	Gene	Relation to gene region	DNA methylation				Gene expression		
			IR	IS	IR − IS	*p* value	IS	IR/IS	*p* value
cg17133045	*AKT3*	Body	0.761 (0.047)	0.735 (0.051)	0.026	0.007	144	0.92	4.38 × 10^−3^
cg04221461	*AKT3*	Body	0.524 (0.070)	0.490 (0.043)	0.034	0.002	144	0.92	4.38 × 10^−3^
cg08428486	*BRAF*	Body	0.807 (0.122)	0.833 (0.035)	−0.026	0.048	235	0.92	3.96 × 10^−4^
cg25204078	*BRAF*	TSS1500	0.771 (0.040)	0.757 (0.042)	0.014	0.034	235	0.92	3.96 × 10^−4^
cg06748146	*HK1*	Body	0.734 (0.047)	0.709 (0.044)	0.026	0.007	170	1.09	3.07 × 10^−3^
cg05514401	*IRS2*	1st exon	0.823 (0.065)	0.792 (0.048)	0.031	0.002	242	0.85	1.24 × 10^−3^
cg18932526	*MAPK8*	TSS1500	0.907 (0.099)	0.929 (0.020)	−0.022	0.020	73	0.94	3.47 × 10^−3^
cg19612574	*MAPK8*	TSS1500	0.935 (0.074)	0.950 (0.019)	−0.015	0.022	73	0.94	3.47 × 10^−3^
cg20994699	*PDX103A*	Body	0.572 (0.092)	0.544 (0.086)	0.028	0.048	100	0.82	4.48 × 10^−4^
cg03465562	*PHKA2*	Body	0.929 (0.092)	0.953 (0.019)	−0.024	0.021	167	0.91	9.80 × 10^−4^

DNA methylation data are expressed as average (SD); gene expression data are expressed as average

IR, insulin-resistant; IS, insulin-sensitive; TSS1500, within 1500 bp of transcriptional start site

### DMS in VAT

CpG methylation in VAT was assessed at 124,089 sites. Although none of the DMS were significant after FDR correction, 10,217 were nominally significant (*p* ≤ 0.05) between insulin-resistant and insulin-sensitive women with median difference in methylation of 0.028 (range 0.001–0.105) (ESM Table 8). We mapped the 10,217 DMS from the present study to other DNA methylation profiling studies in VAT that used the 450 K platform. Benton et al reported 15 DMS in VAT before vs after weight loss induced by bariatric surgery, of which two CpG sites displayed nominally significant and directionally consistent results in the present study (*p* < 0.2) (ESM Table 8) [12]. Guenard et al listed 83 DMS in VAT associated with the metabolic syndrome [24] and, of these, none were differentially methylated in VAT between insulin-resistant and insulin-sensitive women in the present study. Finally, we compared results between SAT and VAT in the present study. Among nominally significant DMS between insulin-resistant and insulin-sensitive women, 1455 CpG sites overlapped between SAT and VAT, 1406 of which displayed directionally consistent results between depots (ESM Table 8).

Next, we merged the 51 differentially associated expressed genes in VAT with the 10,217 DMS and thus identified 18 IR-associated genes containing a total of 29 DMS (Table 6). There were three DMS in two differentially expressed genes that were common between SAT and VAT; cg14229247 (in *ANP32B*), and cg08400424 and cg11796181 (both in *ARHGAP26*) (Table 6). cg14229247 in *ANP32B* could not be confirmed by EpiTYPER, whereas we were unable to design assays for the DMS in *ARHGAP26*, leaving some uncertainty to these results (ESM Table 2). Four genes displayed direct, positive or negative, correlation between gene expression and methylation in VAT (ESM Table 6). Of the 11 DMS in the 5′ region of genes, seven CpG sites displayed an inverse association between gene expression and methylation. Among 18 DMS in gene bodies and 3′UTR regions, two CpG sites displayed coherent changes.

**Table 6:** Differentially expressed genes accompanied by DMS in VAT between insulin-resistant and insulin-sensitive women^a^

Probe	Gene	Relation to gene region	DNA methylation						Gene expression		
			IR		IS		IR − IS	*p* value	IS	IR/IS	*p* value
cg17174775	*AASS*	TSS1500	0.016	0.039	0.027	0.033	−0.011	0.0022	118	0.88	0.000121
cg09711028	*ABCC9*	Body	0.913	0.041	0.897	0.046	0.016	0.033	391	0.86	0.000303
cg16236108	*AGPAT9*	TSS200	0.062	0.042	0.076	0.04	−0.014	0.027	89	0.78	7.62 × 10^−6^
cg14229247^b^	*ANP32B*	TSS1500	0.04	0.048	0.048	0.046	−0.009	0.038	237	0.91	3.61 × 10^−5^
cg08400424^b^	*ARHGAP26*	Body	0.55	0.1	0.597	0.103	−0.047	0.028	109	1.16	2.33 × 10^−5^
cg05185926	*ARHGAP26*	3′UTR	0.712	0.101	0.75	0.121	−0.038	0.025	109	1.16	2.33 × 10^−5^
cg11796181^b^	*ARHGAP26*	Body	0.754	0.043	0.708	0.047	0.046	0.00017	109	1.16	2.33 × 10^−5^
cg12264626	*CA3*	TSS1500	0.183	0.054	0.162	0.095	0.021	0.036	242	0.45	0.000136
cg00908631	*CDKN2C*	TSS1500	0.668	0.052	0.632	0.06	0.036	0.0011	134	0.82	0.000184
cg10156302	*DAPK2*	Body	0.605	0.091	0.552	0.111	0.053	0.01	171	0.86	0.000235
cg23165541	*DAPK2*	5′UTR	0.403	0.094	0.363	0.09	0.039	0.014	171	0.86	0.000235
cg06904649	*DAPK2*	Body	0.767	0.037	0.744	0.043	0.022	0.043	171	0.86	0.000235
cg16151151	*ISL1*	Body	0.196	0.069	0.172	0.095	0.024	0.016	99	0.8	0.000045
cg17686487	*ISL1*	Body	0.434	0.082	0.395	0.089	0.039	0.012	99	0.8	0.000045
cg16270526	*ISL1*	Body	0.225	0.069	0.185	0.077	0.04	0.023	99	0.8	0.000045
cg26422022	*LOX*	TSS200	0.032	0.039	0.039	0.036	−0.007	0.040419	169	1.22	9.74 × 10^−5^
cg22836153	*LOX*	Body	0.057	0.038	0.065	0.033	−0.008	0.042	169	1.22	9.74 × 10^−5^
cg03422350	*MOCS1*	Body	0.722	0.068	0.68	0.071	0.042	0.031	205	0.79	9.42 × 10^−5^
cg10791278	*MOCS1*	Body	0.782	0.056	0.737	0.061	0.045	0.0016	205	0.79	9.42 × 10^−5^
cg06023702	*PAIP2B*	TSS200	0.029	0.043	0.04	0.041	−0.011	0.018	114	0.85	5.02 × 10^−5^
cg06241044	*PAIP2B*	5′UTR	0.265	0.052	0.242	0.066	0.023	0.038	114	0.85	5.02 × 10^−5^
cg22999327	*PDE3A*	Body	0.53	0.105	0.485	0.124	0.045	0.028	133	0.82	1.19 × 10^−5^
cg02631767	*PDE3A*	Body	0.875	0.056	0.857	0.058	0.018	0.048	133	0.82	1.19 × 10^−5^
cg04857033	*PHGDH*	Body	0.376	0.078	0.336	0.089	0.04	0.049	135	0.88	0.00039
cg26166935	*PHLPP1*	Body	0.857	0.039	0.837	0.037	0.02	0.03	101	0.91	0.000203
cg03299121	*PNO1*	TSS200	0.055	0.044	0.064	0.041	−0.01	0.0003	53	1.08	0.000385
cg06123940	*RNF125*	TSS1500	0.798	0.044	0.785	0.043	0.012	0.029	109	0.88	0.0004
cg18101249	*RNF125*	Body	0.082	0.049	0.089	0.037	−0.006	0.046	109	0.88	0.0004
cg13849419	*TJP2*	Body	0.509	0.095	0.468	0.106	0.041	0.043	179	0.93	0.000239

DNA methylation data are expressed as average (SD); gene expression data are expressed as average

^a^Differentially expressed genes (10% FDR) accompanied by DMS (*p* < 0.05) in VAT between insulin-resistant and insulin-sensitive women. Groups were compared using Limma and adjusting for BMI (gene expression, DMS) and age (DMS)

^b^DMS and differentially expressed gene common to SAT and VAT

### DMS in PBMCs

We investigated whether IR was associated with systemic epigenetic differences by analysing DNA methylation profiles in PBMCs. There were no significant DMS after correction for multiple testing among the 99,462 analysed CpG sites, although 2451 were nominally significant with median differences in methylation of 0.021 (range 7 × 10^−5^–0.130) between groups (*p* ≤ 0.05) (ESM Table 9). There were 268 DMS that overlapped between SAT and PBMCs, of which 109 displayed directionally consistent results (ESM Table 4). Among DMS accompanied by differential gene expression in SAT, only three CpG sites displayed significant differential methylation in a consistent direction in PBMCs: *ADAMTS2* cg26694831, average difference in β value between the insulin-resistant and insulin-sensitive women in SAT −0.037 and PBMCs −0.044 (*p* = 0.005), respectively; *FIP1L1* cg19408398, average difference in SAT 0.026 and PBMCs 0.034 (*p* = 0.012), respectively; *SAMD4A* cg06633081 average difference in SAT −0.033 and PBMCs −0.027 (*p* = 0.048), respectively. EpiTYPER analyses of these CpG sites in SAT were non-significant, although DMS in *ADAMTS2* and *FIP1L1* remained directionally consistent (ESM Table 2).

### Cell-mixture-adjusted analysis of DMS

We applied a reference-free algorithm for cell-mixture adjustment to detect DMS, and compared the results with our original whole-tissue-based results [25]. There were 2669, 14,410, and 949 DMS in SAT, VAT and PBMCs, respectively, after cell-mixture adjustment. The number of DMS overlapping between the cell-mixture-adjusted analysis and our original analysis was 948 for SAT, 2059 for VAT and 380 for PBMCs; of these 943, 1999 and 379 DMS, respectively, displayed directionally consistent results (ESM Tables 10–12).

## Discussion

Previous studies have linked WAT CpG methylation to adiposity and type 2 diabetes. Here, for the first time we report a comprehensive analysis of IR-associated DMS and their correlation with gene expression in SAT and VAT.

VAT mass is more strongly associated with IR than SAT, as reviewed [26]. In our genome-wide transcriptome analysis, however, there were a greater number of genes that were differentially expressed in SAT than in VAT in the insulin-resistant state. Nevertheless, the majority of the IR-associated genes displayed differences in expression that were directionally consistent between SAT and VAT. Together, these data suggest that there is no depot-specific transcriptomic signature that is associated with systemic IR. In agreement with this, Klimcáková et al reported similar alterations in the two adipose depots of obese patients with unfavourable metabolic status [27]. This suggests that other factors, such as the amount of VAT or the metabolite profile, could be more important for determining the effect of VAT on IR or other metabolic disorders. We confirm that IR-associated genes in WAT are over-represented for pathways related to immune response and angiogenesis (VEGFR signalling in the present study), whereas reported over-representation of genes important for cell cycle regulation and metabolism was not observed [21, 22]. The reason for the latter discrepancy could be due to selection of study participants.

There were no global differences in DNA methylation between the insulin-resistant and insulin-sensitive women in any of the studied tissues. A number of genes in both SAT and VAT displayed differential methylation accompanied by differential gene expression in insulin-resistant as compared with insulin-sensitive women. We did not observe any significant DMS between the insulin-resistant and insulin-sensitive groups after adjustment for multiple testing in the present dataset. However, considering all nominally significant DMS in the present study (which admittedly include false-positives), the vast majority of DMS that overlap between the present study and previous studies of BMI or type 2 diabetes display directionally consistent methylation differences in the reported cohorts. Furthermore, of the DMS analysed by EpiTYPER, seven had been previously reported and they were all confirmed. This observation suggests that many DMS are real, despite not reaching formal statistical significance in the present study. Traditionally, methylation of CpG islands in promoters has been associated with repression of gene expression whereas CpG sites in gene bodies often display a positive association between methylation and expression [23]. In the present study there was no evidence that DNA methylation in the 5′ regions of genes preferentially repressed gene expression, nor the opposite in gene bodies. Interestingly, the link between transcriptional repression and DNA methylation is less clear for non-CpG island promoters (CpG-poor promoters); many active genes have methylated CpG-poor promoters [28]. Together, the above findings suggest that the relationship between CpG methylation and IR is complex, comprising many CpG sites that have a modest association with IR and a variable impact on gene expression.

There were 223 IR-associated genes with DMS in SAT that were over-represented for pathways related to integrin cell surface interactions, focal adhesion and insulin signalling. Integrins constitute a component of the extracellular matrix and previously have been implicated in adipose remodelling in conjunction with obesity and IR [29, 30]. Specific IR-associated genes with DMS are listed in Table 7, together with potential mechanisms that could explain their association with insulin sensitivity (details on CpG methylation are given in Table 4). These specific genes all have DMS that confirm previous findings, and are associated with adipose tissue and insulin signalling in the literature according to PubMatrix (http://pubmatrix.grc.nia.nih.gov/, accessed 31 August 2015).

**Table 7:** Selected IR-associated genes with DMS

Gene	Expression and CpG-methylation in SAT: observations from the current study	Previously reported findings of gene/protein function
*GAB1*	SAT CpG methylation in the gene body was inversely associated with gene expression and IR was associated with lower *GAB1* expression (fold change IR vs IS: 0.89)	GAB1 is an adaptor molecule that can stimulate adipocyte glucose uptake through a GAB1/PI 3-kinase/PKB/AS160 pathway [31]
*PFKFB3*	SAT CpG methylation in the promoter was directly associated with gene expression, whilst CpG methylation in the gene body was inversely associated. IR was associated with lower *PFKFB3* expression (fold change IR vs IS: 0.80)	PFKFB3 regulates the steady-state concentration of fructose-2,6-bisphosphate, a potent activator of a key regulatory enzyme of glycolysis. Fat cell overexpression of PFKFB3 enhances insulin sensitivity [32]
*IRS2*	SAT CpG methylation in the 5′ region was inversely associated with gene expression and IR was associated with lower *IRS2* expression (fold change IR vs IS: 0.85)	IRS2 mediates the effects of insulin on glucose homeostasis and cell growth
*PTPRJ*	SAT CpG methylation in the gene body was inversely associated with gene expression and IR was associated with higher *PTPRJ* expression (fold change IR vs IS: 1.18)	Recently it was shown that high-fat diet fed *Ptprj* ^−/−^ mice displayed enhanced insulin sensitivity and improved glucose tolerance, thus establishing PTPRJ as a negative regulator of insulin signalling [33]

AS160, Akt substrate 160-KD; GAB1, growth factor receptor bound protein 2-associated binding protein 1; PFKFB3, 6-phosphofructo-2-kinase/fructose-2,6-biphosphatase 3; PI 3-kinase, phosphatidylinositol 3-kinase; PKB, protein kinase B; PTPRJ, protein-tyrosine phosphatases, receptor-type, J

Although, overall, the CpG methylation in PBMCs did not mirror DMS in SAT associated with IR, a few DMS accompanied by differential gene expression in SAT displayed significant differential methylation in a direction consistent with that in PBMCs. CpG methylation results for *FIP1L1* and *ADAMTS2* remained directionally consistent in validation experiments. *FIP1L1* which encodes FIP 1-like, primarily characterised as a fusion protein (*FIP1L1*-*PDGFRA*) in hypereosinophilic disorders [34]. *ADAMTS2* encodes procollagen I N-proteinase that excises the N-propeptide of type I and type II procollagens. Mutation in *ADAMTS2* causes the connective tissue disease Ehlers–Danlos syndrome. None of these genes have been characterised in relation to insulin sensitivity. Neither PBMC, SAT nor VAT DNA methylation signatures could confirm the previously reported association of global leucocyte DNA methylation with IR [35]. Furthermore, a DMS in the *ABCG1* gene in T cells that previously has been associated with HOMA-IR was not detected in the present study [13]. In most cases differences in both gene expression and DNA methylation between groups in the present study were small. One reason for the small differences in DNA methylation could be that DNA from adipose tissue, which contains different cell types having potentially different DNA methylation signatures, were studied. Similarly we investigated unfractionated PBMCs, and the DNA methylation pattern in subpopulations of these cells may differ [9].

There are sex differences in insulin sensitivity [36] and since we only investigated women it is unknown at present whether DNA methylation may have a different role for IR in obese men.

## Conclusion

Whereas global DNA CpG methylation in adipose tissue is not associated with systemic IR, specific genes display differential expression in SAT accompanied by DMS. Such genes include *GAB1*, *IRS2*, *PFKFB3*, and *PTPRJ*. Further analysis of the function and epigenetic regulation of these genes in fat cells will help determine their potential causal role in systemic IR. CpG methylation in PBMCs does not reflect DMS in WAT, suggesting that epigenetic analyses in circulating leucocytes are not suitable for metabolic phenotyping of obese individuals.

## Electronic supplementary material

Below is the link to the electronic supplementary material.

ESM (PDF 465 kb)

ESM Table 1 (XLSX 122 kb)

ESM Table 2 (XLSX 16 kb)

ESM Table 4 (XLSX 1575 kb)

ESM Table 5 (XLSX 75 kb)

ESM Table 7 (XLSX 16 kb)

ESM Table 8 (XLSX 973 kb)

ESM Table 9 (XLSX 292 kb)

ESM Table 10 (XLSX 223 kb)

ESM Table 11 (XLSX 966 kb)

ESM Table 12 (XLSX 83 kb)

## Abbreviations

CpG: CG dinucleotides
DMS: Differentially methylated sites
FDR: False discovery rate
IR: Insulin resistance
PBMC: Peripheral blood mononuclear cell
qPCR: Quantitative real-time PCR
SAT: Subcutaneous adipose tissue
TRAIL: TNF-related apoptosis-inducing ligand
UTR: Untranslated region
VAT: Visceral adipose tissue
VEGFR: Vascular endothelial growth factor receptor
WAT: White adipose tissue

## References

[1] Langenberg C, Sharp SJ, Schulze MB (2012). Long-term risk of incident type 2 diabetes and measures of overall and regional obesity: the EPIC-InterAct case-cohort study. PLoS Med.

[2] Vaag A, Lehtovirta M, Thye-Ronn P, Groop L (2001). Metabolic impact of a family history of Type 2 diabetes. Results from a European multicentre study (EGIR). Diabet Med.

[3] Landin K, Lonnroth P, Krotkiewski M, Holm G, Smith U (1990). Increased insulin resistance and fat cell lipolysis in obese but not lean women with a high waist/hip ratio. Eur J Clin Investig.

[4] Lam TK, Yoshii H, Haber CA (2002). Free fatty acid-induced hepatic insulin resistance: a potential role for protein kinase C-delta. Am J Physiol Endocrinol Metab.

[5] Ravussin E, Smith SR (2002). Increased fat intake, impaired fat oxidation, and failure of fat cell proliferation result in ectopic fat storage, insulin resistance, and type 2 diabetes mellitus. Ann N Y Acad Sci.

[6] Xu H, Barnes GT, Yang Q (2003). Chronic inflammation in fat plays a crucial role in the development of obesity-related insulin resistance. J Clin Invest.

[7] Ravelli AC, van der Meulen JH, Michels RP (1998). Glucose tolerance in adults after prenatal exposure to famine. Lancet.

[8] Gluckman PD, Hanson MA, Buklijas T, Low FM, Beedle AS (2009). Epigenetic mechanisms that underpin metabolic and cardiovascular diseases. Nat Rev Endocrinol.

[9] Reinius LE, Acevedo N, Joerink M (2012). Differential DNA methylation in purified human blood cells: implications for cell lineage and studies on disease susceptibility. PLoS One.

[10] Nilsson E, Jansson PA, Perfilyev A (2014). Altered DNA methylation and differential expression of genes influencing metabolism and inflammation in adipose tissue from subjects with type 2 diabetes. Diabetes.

[11] Agha G, Houseman EA, Kelsey KT, Eaton CB, Buka SL, Loucks EB (2014). Adiposity is associated with DNA methylation profile in adipose tissue. Int J Epidemiol.

[12] Benton MC, Johnstone A, Eccles D (2015). An analysis of DNA methylation in human adipose tissue reveals differential modification of obesity genes before and after gastric bypass and weight loss. Genome Biol.

[13] Hidalgo B, Irvin MR, Sha J (2014). Epigenome-wide association study of fasting measures of glucose, insulin, and HOMA-IR in the Genetics of Lipid Lowering Drugs and Diet Network study. Diabetes.

[14] Bonora E, Targher G, Alberiche M (2000). Homeostasis model assessment closely mirrors the glucose clamp technique in the assessment of insulin sensitivity: studies in subjects with various degrees of glucose tolerance and insulin sensitivity. Diabetes Care.

[15] Lofgren P, Andersson I, Adolfsson B (2005). Long-term prospective and controlled studies demonstrate adipose tissue hypercellularity and relative leptin deficiency in the postobese state. J Clin Endocrinol Metab.

[16] Zhang B, Kirov S, Snoddy J (2005). WebGestalt: an integrated system for exploring gene sets in various biological contexts. Nucleic Acids Res.

[17] Dahlman I, Sinha I, Gao H (2015). The fat cell epigenetic signature in post-obese women is characterized by global hypomethylation and differential DNA methylation of adipogenesis genes. Int J Obes (Lond).

[18] Smyth GK, Gentleman RCV, Dudoit S, Irizarry R, Huber W (2005). Limma: linear models for microarray data. Bioinformatics and computational biology solutions using R and Bioconductor.

[19] Fraga MF, Ballestar E, Paz MF (2005). Epigenetic differences arise during the lifetime of monozygotic twins. Proc Natl Acad Sci U S A.

[20] Rönn T, Volkov P, Gillberg L (2015). Impact of age, BMI and HbA1c levels on the genome-wide DNA methylation and mRNA expression patterns in human adipose tissue and identification of epigenetic biomarkers in blood. Hum Mol Genet.

[21] Elbein SC, Kern PA, Rasouli N, Yao-Borengasser A, Sharma NK, Das SK (2011). Global gene expression profiles of subcutaneous adipose and muscle from glucose-tolerant, insulin-sensitive, and insulin-resistant individuals matched for BMI. Diabetes.

[22] Qatanani M, Tan Y, Dobrin R (2013). Inverse regulation of inflammation and mitochondrial function in adipose tissue defines extreme insulin sensitivity in morbidly obese patients. Diabetes.

[23] Ball MP, Li JB, Gao Y (2009). Targeted and genome-scale strategies reveal gene-body methylation signatures in human cells. Nat Biotechnol.

[24] Guenard F, Tchernof A, Deshaies Y (2014). Differential methylation in visceral adipose tissue of obese men discordant for metabolic disturbances. Physiol Genomics.

[25] Houseman EA, Molitor J, Marsit CJ (2014). Reference-free cell mixture adjustments in analysis of DNA methylation data. Bioinformatics.

[26] Tchernof A, Despres JP (2013). Pathophysiology of human visceral obesity: an update. Physiol Rev.

[27] Klimcáková E, Roussel B, Marquez-Quinones A (2011). Worsening of obesity and metabolic status yields similar molecular adaptations in human subcutaneous and visceral adipose tissue: decreased metabolism and increased immune response. J Clin Endocrinol Metab.

[28] Issa JP, Just W (2011). Epigenetics. FEBS Lett.

[29] Spencer M, Yao-Borengasser A, Unal R (2010). Adipose tissue macrophages in insulin-resistant subjects are associated with collagen VI and fibrosis and demonstrate alternative activation. Am J Physiol Endocrinol Metab.

[30] Henegar C, Tordjman J, Achard V (2008). Adipose tissue transcriptomic signature highlights the pathological relevance of extracellular matrix in human obesity. Genome Biol.

[31] Bertola A, Bonnafous S, Cormont M (2007). Hepatocyte growth factor induces glucose uptake in 3T3-L1 adipocytes through A Gab1/phosphatidylinositol 3-kinase/Glut4 pathway. J Biol Chem.

[32] Huo Y, Guo X, Li H (2012). Targeted overexpression of inducible 6-phosphofructo-2-kinase in adipose tissue increases fat deposition but protects against diet-induced insulin resistance and inflammatory responses. J Biol Chem.

[33] Kruger J, Brachs S, Trappiel M (2015). Enhanced insulin signaling in density-enhanced phosphatase-1 (DEP-1) knockout mice. Mol Metab.

[34] Cools J, Stover EH, Wlodarska I, Marynen P, Gilliland DG (2004). The FIP1L1-PDGFRα kinase in hypereosinophilic syndrome and chronic eosinophilic leukemia. Curr Opin Hematol.

[35] Burghardt KJ, Goodrich JM, Dolinoy DC, Ellingrod VL (2015). DNA methylation, insulin resistance and second-generation antipsychotics in bipolar disorder. Epigenomics.

[36] Geer EB, Shen W (2009). Gender differences in insulin resistance, body composition, and energy balance. Gend Med.

