# Supplementary material for: The epigenetic signature of systemic insulin resistance in obese women
Source: Diabetologia. 2016 Aug 18;59(11):2393–405. doi: 10.1007/s00125-016-4074-5 (PMC5506095; doi:10.1007/s00125-016-4074-5)
Supplement: Supplementary file 1 — (PDF 465 kb) [file 125_2016_4074_MOESM1_ESM.pdf]

Electronic Supplementary Methods

Global Transcriptome assay

WAT specimens (100 mg) were disrupted mechanically and RNA isolated using the RNeasy kit (cat no. 217004, Qiagen) according to the manufacturer's instructions. From high-quality total RNA we prepared and hybridized biotinylated complementary RNA to GeneChip Human Trancriptome Arrays 2.0 (HTA), and then washed, stained and scanned the arrays using standardized protocols (Affymetrix Inc., Santa Clara, CA, USA). There were no replicates in the microarray experiment. Group assignment was blinded during the above experiments. Subsequent data analyses were performed using the Omicsoft ArrayStudio v7.2 (http://www.omicsoft.com/array-studio/). The Robust Multi-array analysis algorithm was used for data normalization and calculation of gene expression. To allow comparisons of transcript levels between samples, all samples were subject to an all-probeset scaling-to-target signal of 100.

Among the 70,523 probesets on the HTA array we first filtered for the 23,442 probesets annotated with a gene symbol. Of the 23,442 probes, 5,860 (25%) transcripts with the lowest mean expression and 5,860 (25%) with the lowest variation in expression, i.e. standard deviation divided by mean expression, were excluded resulting in 11,722 probesets being taken forward for subsequent analysis of differentially expressed genes. The applied cutoff for mean expression will exclude a set of organ-specific genes that should not be expressed in adipose tissue according to the literature. When multiple probesets represented the same gene, we show results for the probeset with the highest call; this does not affect the results (significant p and fold change in expression). Webgestalt was used to identify pathways over-represented among differentially expressed genes and DMS [1].

DNA methylation microarray assays

Genomic DNA was prepared from adipose tissue specimens and from PBMCs using the QiAamp DNA Mini kit as described (cat no. 51304, Qiagen, Hilden, Germany) [2]. The DNA purity and quality was confirmed by an A260/280 ratio >1.8 on a Nanodrop ND-1000 Spectrophotometer (Thermo Fisher Scientific Inc., Waltham, MA, US). The DNA concentration was measured by

Qubit (Life technologies, Stockholm, Sweden). One SAT and one VAT sample were excluded due to insufficient DNA quality.

DNA extracted from SAT and VAT pieces, as well as in PBMCs, was assayed using the Infinium Human Methylation 450 (450K) BeadChips as described (Illumina, San Diego, CA, USA) [2]. Genomic DNA (500 ng) was bisulfite treated using the EZ DNA methylation kit (Zymo Research, Orange, CA, USA) with the alternative incubation conditions recommended when using the Infinium Methylation Assay. The methylation assay was performed on 4 μl bisulfite-converted genomic DNA (50 ng/μl) according to the Infinium HD Methylation Assay protocol (Part #15019519, Illumina). There were no replicates in the microarray experiment. Group assignment was blinded during the above experiments.

BeadChip images were analyzed as described captured using the Illumina iScan. The raw methylation score for each probe represented as a methylation beta-value was calculated using the GenomeStudio Methylation module software (2010.3) [3]. All included samples showed high quality bisulfite conversion according to Zymo-control samples and also passed all GenomeStudio quality control steps based on built in control probes for staining, hybridization, extension and specificity. We used the Bioconductor Lumi package to perform color and quantitative normalization of the DNA methylation data [4]. The BMIQ package was used to adjust the beta-values of type 2 design microarray probes into a statistical distribution characteristic of type 1 probes [5]. For differential methylation analysis, beta-values were converted to M-values [M = log2(beta/(1-beta))], which have a more appropriate distribution for statistical tests of comparisons between groups. As beta-values are easier to interpret biologically, beta-values are retained when describing the results. Adjustment for array plate and bisulfite treatment batch was performed using ComBat [6].

The Infinium Human Methylation 450 BeadChip array contains 485,577 probes, which covers 21,231 RefSeq genes. Before analysis of differentially methylated sites (DMS) a number of filtering steps were performed. Probes containing common SNPs with minor allele frequency (MAF) >10% or with SNPs within 10 basepairs from the interrogated CpG sites according to Illumina file "humanmethylation450_dbsnp137.snpupdate.table.v2.sorted" were excluded leaving 319,569 probes for subsequent analysis. Next, non-specific probes, i.e. hybridizing to $\geq$2 sites, were excluded leaving 302,822 probes for the next step [7]. In analysis of DMS we further

excluded probes not annotated to a gene according to Illumina leaving 236,147 probes. Finally, in each tissue separately we filtered to include only the probes that passed the threshold variance 0.1 in beta-value (Qlucore, www.qlucore.com); thus, 112,057 (SAT), 124,089 (VAT) and 99,462 (PBMCs) probes, respectively, were taken forward to identify DMS.

Validation experiments

Quantitative methylation analysis was performed using the EpiTYPER methodology [8] and the MassARRAY ® system (Agena Biosciences, San Diego, CA, USA) according to manufacturer's recommendations and protocols. In the method a targeted amplification of bisulfite converted DNA is followed by in vitro transcription, RNase cleavage and subsequent fragment mass analysis by Matrix-Assisted Laser Desorption/Ionization Time of Flight Mass Spectrometry (MALDI-TOF MS) to quantify CpG sites.

PCR primers for amplicons encompassing the 450K cg:s of interest were designed using EpiDesigner (Agena Biosciences) and checked for bisulfitome specificity by using BiSearch in silico PCR (http://bisearch.enzim.hu/?m=genompsearch). A 4-point dilution curve (0% methylated, 25% methylated, 50% methylated and 100% methylated) of EpiTect methylated and non-methylated bisulfite treated control DNA (Qiagen) was used to evaluate the quantitative recapture of methylation ratios of the amplicons. The 4-point dilution curve was run in triplicate and also provided data for standard deviation analysis. The amplicons used in this study all met the quality criteria of fully methylated and non-methylated data points measured at > 75% and < 20% methylation ratios, respectively, as well as standard deviation percentages < 10%. Samples were run in duplicate and standard deviation percentages >20% were removed from the study. The remaining data points correlated with R^2 0.99. Bisulfite conversion efficiency in all samples was evaluated by analyzing 13 non-CpG C:s spread out in the amplicons analyzed in the study. All data was checked by manually and visually inspecting the mass spectra. Group assignment was blinded during the above experiments.

References

[1] Zhang B, Kirov S, Snoddy J (2005) WebGestalt: an integrated system for exploring gene sets in various biological contexts. Nucleic Acids Res 33: W741-748

[2] Dahlman I, Sinha I, Gao H, et al. (2015) The fat cell epigenetic signature in post-obese women is characterized by global hypomethylation and differential DNA methylation of adipogenesis genes. Int J Obes (Lond)

[3] Bibikova M, Lin Z, Zhou L, et al. (2006) High-throughput DNA methylation profiling using universal bead arrays. Genome research 16: 383-393

[4] Du P, Kibbe WA, Lin SM (2008) lumi: a pipeline for processing Illumina microarray. Bioinformatics 24: 1547-1548

[5] Teschendorff AE, Marabita F, Lechner M, et al. (2013) A beta-mixture quantile normalization method for correcting probe design bias in Illumina Infinium 450 k DNA methylation data. Bioinformatics 29: 189-196

[6] Johnson WE, Li C, Rabinovic A (2007) Adjusting batch effects in microarray expression data using empirical Bayes methods. Biostatistics 8: 118-127

[7] Chen YA, Lemire M, Choufani S, et al. (2013) Discovery of cross-reactive probes and polymorphic CpGs in the Illumina Infinium HumanMethylation450 microarray. Epigenetics 8: 203-209

[8] Ehrich M, Nelson MR, Stanssens P, et al. (2005) Quantitative high-throughput analysis of DNA methylation patterns by base-specific cleavage and mass spectrometry. Proc Natl Acad Sci U S A 102: 15785-15790

ESM Table 3. Differentially expressed Insulin Pathway genes in SAT between insulin resistant and sensitive women

Gene		IS average	IR/IS
ABCC1	ATP-binding cassette, sub-family C (CFTR/MRP), member 1	189	1.13
ACTA1	actin, alpha 1, skeletal muscle	197	1.07
ADCY7	adenylate cyclase 7	178	1.08
ADRB2	adrenoceptor beta 2, surface	146	0.89
AKT3	v-akt murine thymoma viral oncogene homolog 3 (protein kinase B, gamma)	144	0.92
ARHGAP26	Rho GTPase activating protein 26	111	1.09
ARNT	aryl hydrocarbon receptor nuclear translocator	302	0.94
BCL2L1	BCL2-like 1	151	1.07
BMP6	bone morphogenetic protein 6	307	0.91
BMPR2	bone morphogenetic protein receptor, type II (serine/threonine kinase)	513	0.92
BNIP3	BCL2/adenovirus E1B 19kDa interacting protein 3	299	0.92
BNIP3L	BCL2/adenovirus E1B 19kDa interacting protein 3-like	966	0.93
BRAF	v-raf murine sarcoma viral oncogene homolog B1	235	0.92
CCND2	cyclin D2	675	1.21
CCNG1	cyclin G1	295	0.88
CITED2	Cbp/p300-interacting transactivator, with Glu/Asp-rich carboxy-terminal domain, 2	333	0.86
CSF1	colony stimulating factor 1 (macrophage)	219	1.12
CSK	c-src tyrosine kinase	110	1.06
CSRP2	cysteine and glycine-rich protein 2	174	0.82
CUL1	cullin 1	274	0.95
CYTH1	cytohesin 1	141	1.06
DDIT4	DNA-damage-inducible transcript 4	234	1.17
EIF4B	eukaryotic translation initiation factor 4B	815	0.89
EPS15	epidermal growth factor receptor pathway substrate 15	498	0.92
FBXO11	F-box protein 11	204	0.91
FBXO32	F-box protein 32	107	0.86
FRS2	fibroblast growth factor receptor substrate 2	155	0.93
GAB1	GRB2-associated binding protein 1	141	0.89
GNAI1	guanine nucleotide binding protein (G protein), alpha inhibiting activity polypeptide 1	310	0.85
HBP1	HMG-box transcription factor 1	235	0.91
HK1	hexokinase 1	170	1.09
IL6R	interleukin 6 receptor	133	1.07
IRF8	interferon regulatory factor 8	205	1.29
IRS2	insulin receptor substrate 2	242	0.85

ITGA1	integrin, alpha 1	461	0.87
JMY	junction mediating and regulatory protein, p53 cofactor	128	0.94
KDR	kinase insert domain receptor (a type III receptor tyrosine kinase)	313	0.85
KLF8	Kruppel-like factor 8	219	0.90
MAP3K5	mitogen-activated protein kinase kinase kinase 5	132	0.83
MAPK8	mitogen-activated protein kinase 8	73	0.94
NBN	nibrin	174	0.93
NDRG1	N-myc downstream regulated 1	506	0.92
NEDD4L	neural precursor cell expressed, developmentally down-regulated 4-like, E3 ubiquitin protein ligase	185	0.80
NR3C1	nuclear receptor subfamily 3, group C, member 1 (glucocorticoid receptor)	248	0.91
NRIP1	nuclear receptor interacting protein 1	518	0.91
PAG1	phosphoprotein associated with glycosphingolipid microdomains 1	70	1.10
PAWR	PRKC, apoptosis, WT1, regulator	122	0.87
PDE3B	phosphodiesterase 3B, cGMP-inhibited	673	0.86
PFKFB3	6-phosphofructo-2-kinase/fructose-2,6-biphosphatase 3	542	0.80
PKM	pyruvate kinase, muscle	320	1.18
PPM1A	protein phosphatase, Mg2+/Mn2+ dependent, 1A	156	0.94
PTPRJ	protein tyrosine phosphatase, receptor type, J	139	1.18
RANBP9	RAN binding protein 9	321	0.89
RB1CC1	RB1-inducible coiled-coil 1	165	0.89
RBL2	retinoblastoma-like 2 (p130)	307	0.92
RICTOR	RPTOR independent companion of MTOR, complex 2	148	0.92
SERPINE1	serpin peptidase inhibitor, clade E (nexin, plasminogen activator inhibitor type 1), member 1	69	1.17
SIN3A	SIN3 transcription regulator homolog A (yeast)	251	0.94
SIRT1	sirtuin 1	175	0.91
SLC3A2	solute carrier family 3 (activators of dibasic and neutral amino acid transport), member 2	198	1.07
SMAD5	SMAD family member 5	186	0.93
SMAD9	SMAD family member 9	104	0.94
SMARCC2	SWI/SNF related, matrix associated, actin dependent regulator of chromatin, subfamily c, member 2	488	0.93
TBL1XR1	transducin (beta)-like 1 X-linked receptor 1	379	0.94
TJP1	tight junction protein 1	453	0.89
UBE3A	ubiquitin protein ligase E3A	208	0.93
USP8	ubiquitin specific peptidase 8	211	0.92
WASL	Wiskott-Aldrich syndrome-like	266	0.94
YES1	v-yes-1 Yamaguchi sarcoma viral oncogene homolog 1	189	0.91
YWHAH	tyrosine 3-monooxygenase/tryptophan 5-monooxygenase activation protein, eta polypeptide	205	1.06

ESM Table 6. Direct correlations between CpG methylation and gene expression

SAT[a]

Gene	Probe	Gene region	Expression IR-IS (Log2)	Methylation IR-IS (beta)	Correlation[c]
ABCC3	cg19734752	Body	1.259	0.024	0.192
ADAMTS15	cg19333883	Body	1.203	0.032	0.178
ADAMTS2	cg21183664	Body	1.112	0.031	0.275
ALDH1A1	cg01812894	TSS1500	-1.466	0.030	-0.219
AMPD3	cg19132462	TSS200	1.142	-0.039	-0.101
ATP10A	cg03924551	Body	1.119	0.030	0.313
C1QTNF7	cg24829483	5'UTR	-1.161	0.040	-0.168
C1QTNF7	cg00545229	TSS200	-1.161	0.049	-0.137
C1QTNF7	cg03290977	Body	-1.161	-0.034	0.167
CHST3	cg10772263	5'UTR	1.139	0.049	-0.143
COL5A1	cg14274542	Body	1.101	0.039	0.175
COL5A1	cg24354213	Body	1.101	0.024	0.230
CPED1	cg03464229	Body	-1.162	-0.038	0.247
CYP4X1	cg27291464	TSS1500	-1.236	0.038	-0.290
EDNRA	cg17073859	TSS1500	-1.338	0.037	-0.197
EDNRA	cg00974629	TSS200	-1.338	0.050	-0.297
GPC1	cg02653521	TSS1500	1.107	-0.062	-0.226
IRF8	cg10334323	Body	1.254	0.042	0.302
KCNAB1	cg01800345	Body	-1.138	0.025	0.117
NECAB1	cg23133255	TSS1500	-1.460	0.028	-0.195
NIPSNAP3B	cg13888748	TSS1500	-1.334	0.052	-0.294
PCMTD1	cg00836007	5'UTR	-1.114	0.044	-0.192
PTGER3	cg17888090	Body	-1.146	-0.026	0.199
PTGER3	cg16823292	Body	-1.146	-0.054	0.190
RHOT1	cg06674117	Body	-1.124	-0.055	0.167
RNF217	cg18196453	Body	-1.103	-0.058	0.253
ROR1	cg03225664	Body	1.120	-0.024	0.138

ROR1	cg24052817	Body	1.120	-0.029	0.106
SAMD4A	cg23901920	Body	1.102	0.041	0.201
SAMD4A	cg26437697	Body	1.102	0.040	0.124
SEMA3G	cg07357683	TSS1500	1.189	-0.020	-0.211
SH3PXD2B	cg05223396	Body	1.108	0.034	0.134
SLC4A4	cg07991704	5'UTR	-1.295	0.045	-0.169
SYNE2	cg16725974	5'UTR	-1.129	0.047	-0.228
TSPYL2	cg22170936	TSS200	-1.149	0.040	-0.242
VTRNA1-3	cg23910413	TSS1500	1.152	-0.049	-0.125
**VAT**[b]					
CA3	cg12264626	TSS1500	-1.945	0.029	-0.313
CA3	cg13721134	TSS1500	-1.945	0.028	-0.323
CDKN2C	cg00908631	TSS1500	-1.229	0.048	-0.104
DAPK2	cg23165541	5'UTR	-1.199	0.055	-0.225
PAIP2B	cg06241044	5'UTR	-1.155	0.030	-0.175

a. We calculated correlation between expression of 647 differentially expressed genes and 10,746 DMS in SAT between IR and IS women.

b. We calculated correlation between expression of 51 differentially expressed genes and 10,217 DMS in VAT between IR and IS women.

c. Shown are Spearman correlation coefficient <-0.1 (CpG-sites in TSS1500, TSS200 or 5'UTR) or >0.1 (CpG-sites in gene body or 3'UTR).

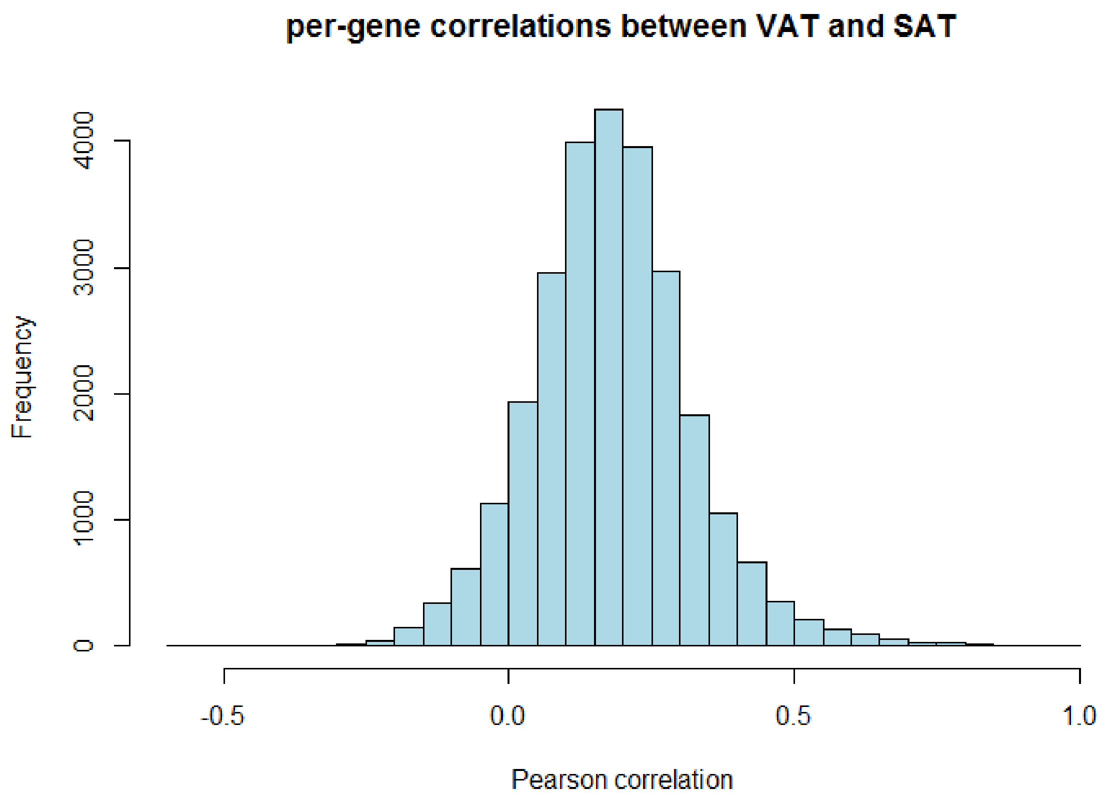

ESM Fig. 1A. Histogram of per-gene correlation between VAT and SAT tissue samples, respectively. Log_2 normalized mRNA expression levels were used to compute the Pearson correlation coefficient for each gene by matching corresponding VAT and SAT tissue samples from each participant.

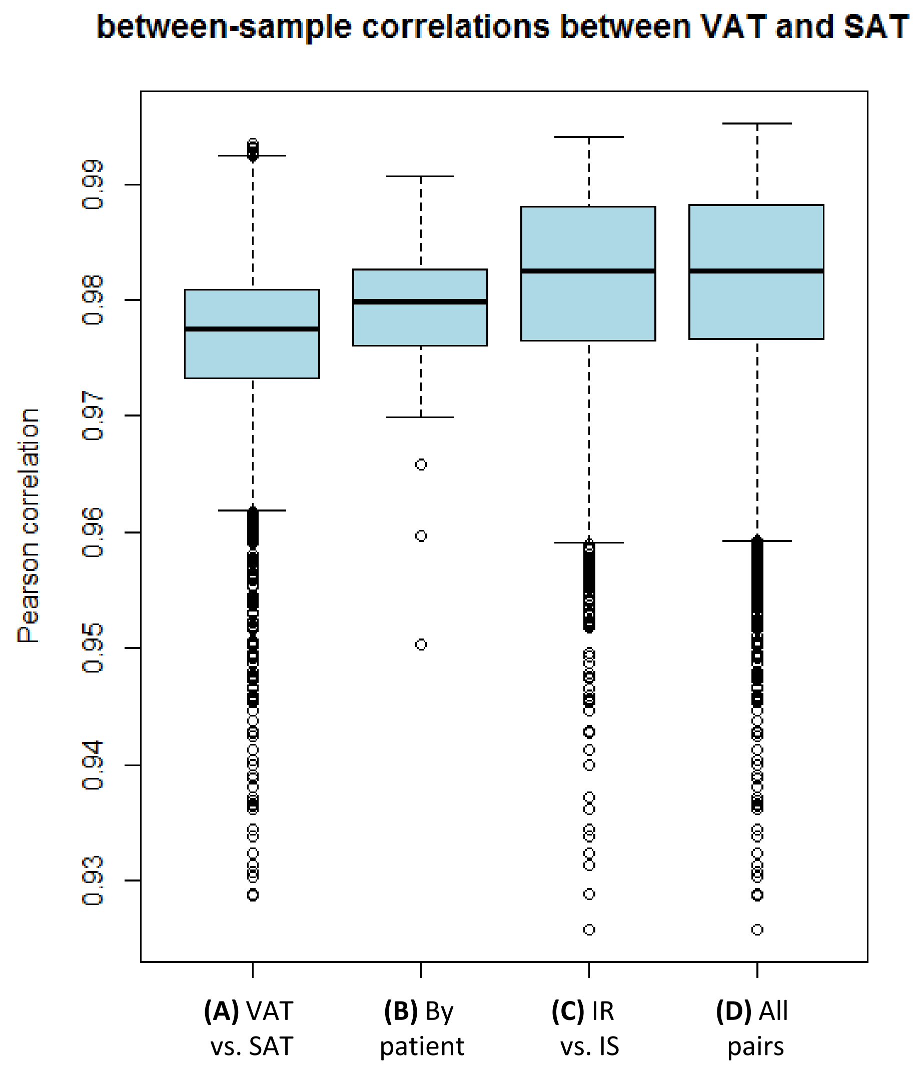

ESM Fig. 1B. Boxplots of between-sample Pearson correlation coefficients. Coefficients were computed using $\log_2$ normalized mRNA expression levels for all genes represented on the array. **A)** The first box shows the correlation between VAT and SAT samples. **B)** Same as A) but showing only the sample pairs from the same participant. As expected, within-participant correlation is higher than between-participant. For comparison, we show **C)** correlation between IR and IS samples, and **D)** all pairwise correlation coefficients. As expected, within-tissue type correlation coefficients are on average higher than between-tissue type comparisons.
